# Supplementary material for: A protocol for recruiting and analyzing the disease-oriented Russian disc degeneration study (RuDDS) biobank for functional omics studies of lumbar disc degeneration
Source: PLoS One. 2022 May 13;17(5):e0267384. doi: 10.1371/journal.pone.0267384 (PMC9106166; doi:10.1371/journal.pone.0267384)
Supplement: S1 File — (DOCX) [file pone.0267384.s001.docx]

## INFORMATION FOR THE PARTICIPANT OF RUSSIAN DISC DEGENERATION STUDY (RuDDS)

| **Name of the study** | Russian Disc Degeneration Study (RuDDS) | |
| --- | --- | --- |
| **Head researcher** | ХХХ ХХХ | |
| **Telephone number of the head researcher, available 24 hours a day** | +7 XXX XXX XX XX | |
| **Name and address of the research center** |  | |
| **Ethics Committee** | **Name** |  |
|  | **Address** |  |
|  | **Telephone** |  |

1. **Invitation**

You are invited to take part in scientific research. In this document you will find a description of Russian Disc Degeneration Study (RuDDS).

Please take the time to read this document carefully and decide if you want to participate in this study or not.

1. **What is the purpose of this study?**

You are invited to take part in this research because you underwent MRI of the lumbar spine and may have clinical symptoms of degenerative disease of the lumbar spine of varying intensity, such as: back pain, pain in the leg/legs, numbness in the leg/legs, and decreased range of motion in the back. The diagnosis of degenerative disease of the lumbar spine could have been made to you earlier, or you first consulted a doctor concerning the above clinical symptoms. To identify the mechanisms of formation and search for the risk factors of initiation and intensification of intervertebral disc degeneration, you are invited to participate in this research.

1. **Do I need to participate in this study?**

You can decide for yourself whether you participate in this research or not (this is your choice). If you choose to participate in the study, you will be asked to complete, sign and date this Patient Information and Consent Form and keep it with you as it contains useful information about the research and doctor's contact numbers. You can still opt out of further participation in the study at any time without explaining the reason, and your decision will not affect the quality of your further treatment. You will be informed immediately if additional information that may affect your consent to continue participating in the research becomes available at any time.

1. **What happens if I agree to participate in this study and what I need to do?**

If you decide to participate in the study, your doctor will collect information about you and your anamnesis. Information about your condition or treatment that may concern with your participation in the study will be collected. As part of this study, your doctor will set up one (1) to three (3) visits for you, depending on your treatment plan.

***Visit 1***

To satisfy the requirements of participating in the research, during your first visit you will have the medical tests and procedures listed below:

- To start participating in the research and before collecting any medical information, your doctor will talk to you and ask you to sign a Consent Form;
- The doctor will ask you to answer questions about complaints, medical history, medicines taken currently and in the past, data on changes in health; and examine the data of the surveys carried out;
- Demographic information, in particular, information about gender, date of birth, age, nationality, marital status, frequency of physical activity, and lifestyle will be collected and documented;
- You will be asked to complete one scale on the severity of back and/or leg pain and one questionnaire on the quality of life and health status before surgery;
- The doctor will review the results of the magnetic resonance tomography of the lumbar spine that you have undergone earlier. These data will help the doctor to determine the causes of pain, nerve compression, position of the vertebrae and determine the tactics of treatment.
- Procedure for taking blood for genetic analysis.

If, based on the results of the procedures performed during Visit 1, you meet the inclusion criteria and do not meet any of the exclusion criteria for this research, a blood sample will be taken. Blood will be taken in an amount of 10-15 ml (approximately 1 tablespoon) for genetic analysis.

If conservative treatment is recommended to you, then at this stage the need for your personal presence ends.

If you are scheduled for surgical treatment, additional procedures will be performed: Visit 2 and Visit 3 (**described below**).

*If necessary, researchers can contact you in addition to the appointed Visits, to clarify any data or to invite you for an additional examination.*

ONLY FOR THOSE PATIENTS WHO ARE SCHEDULED FOR SURGERY

*Visit 2. Surgery*

You will undergo a planned surgical treatment. During this treatment the nerves will be released, information about the performed procedure will be collected. A part of the spinal disc herniation removed during the surgery will be sent for genetic analysis.

*Visit 3. Follow-up studies in 3 months*

During this visit, the following procedures will be performed and the following information will be collected:

- The doctor will ask you to answer questions about your well-being, possible adverse events, and taken anaesthetics.
- You will be asked to complete one scale on the severity of back and/or leg pain and one questionnaire on the quality of life and health status in comparison with preoperative time.

The research doctor will ask if you are currently working and assess your recovery and overall health.

*This information can be collected remotely in the form of a telephone interview.*

1. **What are possible disadvantages and types of risks?**

The usual clinical examination will be somewhat expanded due to a more detailed survey of your demographic data. Additional blood collection for genetic analysis has the same risks as taking blood for a regular clinical analysis, namely: mild and moderate pain during a vein puncture is possible, a hematoma (bruise) may appear at the injection site; in rare cases, there can be edema and redness. If you have noticed any of these signs, tell your doctor about it. In the event that you undergo surgical treatment, taking the surgical material (a fragment of a disc herniation) for genetic analysis will not bring you any additional unpleasant sensations, since it does not affect the procedure of surgical intervention. The rest of the study protocol is performed in the framework of standard healthcare.

1. **What are possible benefits of participating in the study?**

If you agree to participate in this research, it is likely that you will not receive any direct medical benefits. Your examination and treatment before and after blood collection and surgical treatment will be performed in accordance with the standards of healthcare and local practice, whether you are participating in the study or not. However, your participation in this research will help to identify the risk factors for degeneration of the lumbar spine discs, to determine the mechanisms of their occurrence. Further tactics of your treatment can be planned considering the revealed patterns.

1. **Are there any alternative treatment approaches?**

You are not obliged to participate in this study to be monitored and treated for degenerative lumbar spine disease. If you refuse to participate in this research, you will receive standard medical care at the discretion of your doctor. You can also refuse surgery and choose conservative treatment, which will be recommended by a neurologist on the outpatient admission.

1. **Whether my participation in the study is confidential?**

Yes, all information obtained from your medical record, medical history will be analyzed anonymously, treated as confidential information and stored in a secure computer file. Representatives of Health Regulators and Ethics Committee may have confidential access to your medical records and files. If necessary, your doctor responsible for the study may contact your relatives or friends, as well as the attending physician or other medical personnel responsible for your treatment, to collect information about your health condition, if this is important for this research.

You will have the right to have access to your personal information and to make corrections through your doctor responsible for the study.

In the event of premature termination of participation in the research, all information obtained up to that point will be used.

1. **How the study results will be used?**

The results of this research will be published in a medical journal and/or transferred to regulatory organizations. The information will be treated as confidential and under no circumstances will your name be disclosed.

1. **Insurance and participation cost**

Physical examinations, genetic tests, and other procedures performed as part of this research will be free of charge to you. There is no material reward for participating in the study.

1. **Who has evaluated this study?**

The protocol of this research has been reviewed and approved by the Local Ethics Committee of the Federal State Budgetary Institution “Novosibirsk Research Institute of Traumatology and Orthopedics named after Y. L. Tsivyan” of the Russian Ministry of Health. If you have any questions about your rights as a participant, you should contact the secretary of the local Ethics Committee center XXX XXX by phone: XXXXXXXXXXX.

**Contact information**

If you have any questions about this research or about your participating, you should contact Research Coordinator XXX XXX by phone XXX XXX XXXXX.

**Thank you for considering the opportunity of participating in this study.**

**CONSENT FORM**

- I have read and understood the information presented in the Consent Form;
- I was given the opportunity to ask questions to which I received comprehensive and satisfying answers;
- I have received a signed and dated copy of this Consent Form.
- I give voluntary consent to participate in this research until I decide otherwise;
- By signing this document, I am not deprived of my legal rights;
- I agree not to restrict the use of data or results obtained in the course of this research if they are used solely for scientific purposes;
- I understand that even if I end my participation in this study, the research physician and its designates, the ethics committee and the competent authorities will not need my consent to review my medical records for the purposes of this study and for the purposes of any other research that may be conducted in the future and will be associated with it;

I agree to give access to my medical records. However, I understand that my identity will not be disclosed in any form of information published or shared with the third parties.

Patient surname, name, and patronymic name

Patient signature, date

Doctor surname, name, and patronymic name

Doctor signature
