# Supplementary material for: A protocol for recruiting and analyzing the disease-oriented Russian disc degeneration study (RuDDS) biobank for functional omics studies of lumbar disc degeneration
Source: PLoS One. 2022 May 13;17(5):e0267384. doi: 10.1371/journal.pone.0267384 (PMC9106166; doi:10.1371/journal.pone.0267384)
Supplement: Attachment — Submitted filename: Response to Reviewers.docx [file pone.0267384.s003.docx]

Dear Editor,

We would like to thank you and the reviewers for all the helpful comments and suggestions. We have carefully revised the manuscript according to the comments provided by you and the reviewers and added the necessary clarifications and corrections. Please find our point-by-point responses below. We hope that you and the reviewers will find the revised manuscript suitable for publication in Plos ONE.

We include the updated conflict of interest statement: “YSA is a founder and co-owner of PolyOmica and PolyKnomics, private organisations that provide services, research, and development in the field of quantitative and statistical genetics and computational genomics. This does not alter our adherence to PLOS ONE policies on sharing data and materials. Other authors declare no conflicts of interest.”

We would like to emphasize that this manuscript is a study protocol of biobank recruitment and analysis. Since the recruitment is an ongoing process, we do not report any data here. All clinically relevant data, such as genotypic data, medical history, MRI scans, results of transcriptome and N-glycome profiling could not be deposited in open access repositories. Access to this data and accesses to the biological samples could be granted on a collaborative basis upon special request to the data access committee of the Institute of Cytology and Genetics SB RAS. The summary-level data from the analyses (e.g. GWAS results) will be publically available via standard corresponding data sharing platforms (e.g. Zenodo, https://zenodo.org/).

We have reformatted the “Data Access” section accordingly.

Yours Sincerely,

On behalf of the coauthors

Dr. Olga Leonova

-----------------------------

Journal Requirements:

1. Please ensure that your manuscript meets PLOS ONE's style requirements, including those for file naming. The PLOS ONE style templates can be found at

https://journals.plos.org/plosone/s/file?id=wjVg/PLOSOne_formatting_sample_main_body.pdf and

https://journals.plos.org/plosone/s/file?id=ba62/PLOSOne_formatting_sample_title_authors_affiliations.pdf

**A.: We formatted the manuscript according to the requirements.**

2. Thank you for stating the following in the Competing Interests section: "YSA is a co-owner of Maatschap PolyOmica and PolyKnomics BV, private organizations, providing services, research and development in the field of quantitative and computational genomics."

Please confirm that this does not alter your adherence to all PLOS ONE policies on sharing data and materials, by including the following statement: "This does not alter our adherence to PLOS ONE policies on sharing data and materials.” (as detailed online in our guide for authors http://journals.plos.org/plosone/s/competing-interests). If there are restrictions on sharing of data and/or materials, please state these. Please note that we cannot proceed with consideration of your article until this information has been declared.

Please include your updated Competing Interests statement in your cover letter; we will change the online submission form on your behalf.

**A.: We confirm it and added the sentence into Conflict of interest section.**

3. We note that you have stated that you will provide repository information for your data at acceptance. Should your manuscript be accepted for publication, we will hold it until you provide the relevant accession numbers or DOIs necessary to access your data. If you wish to make changes to your Data Availability statement, please describe these changes in your cover letter and we will update your Data Availability statement to reflect the information you provide.

**A.: All clinically relevant data, such as genotypic data, medical history, MRI scans, results of transcriptome and N-glycome profiling could not be deposited in open access repositories. Access to this data and accesses to the biological samples could be granted on a collaborative basis upon special request to the Steering Committee of the Institute of Cytology and Genetics SB RAS. The summary-level data from the analyses (e.g. GWAS results) will be publically available via standard corresponding data sharing platforms (e.g. Zenodo, https://zenodo.org/).**

4. In your Data Availability statement, you have not specified where the minimal data set underlying the results described in your manuscript can be found. PLOS defines a study's minimal data set as the underlying data used to reach the conclusions drawn in the manuscript and any additional data required to replicate the reported study findings in their entirety. All PLOS journals require that the minimal data set be made fully available. For more information about our data policy, please see http://journals.plos.org/plosone/s/data-availability.

Upon re-submitting your revised manuscript, please upload your study’s minimal underlying data set as either Supporting Information files or to a stable, public repository and include the relevant URLs, DOIs, or accession numbers within your revised cover letter. For a list of acceptable repositories, please see http://journals.plos.org/plosone/s/data-availability#loc-recommended-repositories. Any potentially identifying patient information must be fully anonymized.

Important: If there are ethical or legal restrictions to sharing your data publicly, please explain these restrictions in detail. Please see our guidelines for more information on what we consider unacceptable restrictions to publicly sharing data: http://journals.plos.org/plosone/s/data-availability#loc-unacceptable-data-access-restrictions. Note that it is not acceptable for the authors to be the sole named individuals responsible for ensuring data access.

We will update your Data Availability statement to reflect the information you provide in your cover letter.

**A.: Since it is a study protocol, we do not report any data here. The minimal sample size of the study is expected to be 1,100 patients with whole-genome genotyping and total plasma protein N-glycosylation profiles measured for at least 384 participants. Expression profiles of approximately 40 disc specimens will be measured.**

**Summary-level data generated based on omics profiles (e.g. GWAS results) will be made publically available after study completion via standard open sources for the corresponding data type sharing.**

**All clinically relevant data, such as genotypic data, medical history, MRI scans, results of transcriptome and N-glycome profiling could not be deposited in open access repositories due to legal restrictions (data contains sensitive information).**

**Individual-level data, such as medical history, MRI scans, genotypes, results of transcriptome and N-glycome profiling as long as biological specimens will be available upon special formal request to the data access committee of the Institute of Cytology and Genetics SB RAS.**

5. Please include captions for your Supporting Information files at the end of your manuscript, and update any in-text citations to match accordingly. Please see our Supporting Information guidelines for more information: http://journals.plos.org/plosone/s/supporting-information.

[Note: HTML markup is below. Please do not edit.]

**A:. Done.**

**Reviewers' comments:**

Reviewer #1: Thank you for giving me this opportunity to review this article. The article is well written, though I have some serious concerns regarding the article.

Abstract:

1. Mention the study objectives in detail.

2. Include the allocation procedure in detail.

3. Mention the character of study participants.

4. Mention the statistical tests used for the study analysis.

**A.: We rewrote the Abstract removing sectioning to make it fit the PLOS criteria. We have described the allocation details in Materials and Methods.**

Manuscript

1. Include the clinical significance of this study over clinicians, patients, and researchers.

**A.: We added the clinical significance of this study over clinicians, patients, and researchers to the Introduction (see lines 75-80) and Discussion part (see lines 345-352).**

2. Mention the eligibility criteria of the study participants.

**A.: We added a more detailed description of participants of the study to the “Study design and settings” part of the manuscript (see lines 95-97). A detailed description of the eligibility criteria of the study participants had been given in the “Patient selection and expected sample size” section of the Methods part (see lines 119-129). The eligibility criteria are listed in Table 2.**

3. Mention who has diagnosed the condition and is included in the trial?

**A.: We added the description of who has diagnosed the condition of the study participants (see line 121; “Patient selection and expected sample size” section of the Methods part). More precise characteristics of the study participants were mentioned in the “Study design and settings” (see lines 95-97) and “Patient selection and expected sample size” (see lines 119-129) sections of the Methods.**

4. Include the randomization and allocation procedure in detail.

**A.: We included the randomization and allocation procedure description to the Methods part (see lines 179-183, 198-200, 208-210).**

5. Mention the method of sample size calculation with reference.

**A.: We added the description of sample size calculation to “Sample size calculation and power estimation” section of “Statistical data analysis” (lines 231-236).**

6. Mention the statistical tests used for the study analysis.

**A.: We added information on statistical tests to the Methods for further data analysis (see lines 223-228, 267-269).**

7. Summarize the contents of the discussion part.

**A.: We added a summary of the Discussion part (see lines 353-359).**

Reviewer #2:

Title

1. Title needs to be more specific

2. Kindly frame title such that it is accurate, informative, descriptive, succinct, simple and specific.

**A.: We corrected the title to make it more concrete.**

Abstract

1. Key words Better to be added at the end of the abstract.

**A.: We added the key words at the end of the Abstract (see lines 39-40).**

2. Methods section is poorly framed. It has to be re-written.

**A.: We reformatted the Method section according to the comments of both Reviewers.**

Introduction

1. English language need to be edited

**A.: We have corrected English through the text.**

2. Explain the rationale of the study ,Kindly focus on three elements of introduction.

a. What is known about the topic? (Background)

b. What is not known? (The research problem)

c. Why the study was done? (Justification)

**A.: We have reformatted Introduction section according to the comments of both Reviewers.**

Methods

1. Add references to methods section

2. Statistical methods that will be used need to be explained in details

**A.: We have reformatted and updated the Methods section, added the information about statistical tests and methods (see the “Statistical data analysis”) and references.**
